# Numerical analysis of stresses on angular contact ball bearing under the static loading with respect to race thickness and housing stiffness

**DOI:** 10.1038/s41598-024-66479-y

**Published:** 2024-07-19

**Authors:** Bruno R. Mose, Dong-Kil Shin, Jeong-Hwan Nam

**Affiliations:** 1 Department of Mechanical Engineering, Jomo Kenyatta University of Agriculture & Technology, Nairobi, Kenya; 2 School of Mechanical Engineering, Yeungnam University, 280 Daehak-Ro, Gyeongsan, 38541 Gyeongbuk Korea; 3 Department of Mechanical System Engineering, Dongyang University, 145 Dongyangdaero, Punggi, Yeongju, 750-711 Gyeongbuk Korea

**Keywords:** Contact stress, Angular contact ball bearing, Finite element analysis, Bearing failure, Engineering, Materials science

## Abstract

A 3-dimensional model of the angular contact ball bearing (ACBB) was modeled using Abaqus/standard (Dassault systems- version 2017) to investigate the influence of race thickness on the bearing performance. It was found that the ability to support higher contact stress increased with race thickness. However, large deformations were found to occur on outer race with thickness of 3.3 mm and only small deformations were observed on outer race with a thickness of 9.9 mm. The large deformations induce higher shear stresses on thin races than on thick races. These stresses cause spall growth in bearings and propagate into a network of cracks. As a result of these findings, thin races are prone to failure compared with thick races.

## Introduction

Contact stress is one of the key factors that influence bearing life. High contact stresses can contribute to crack initiation and spall growth in bearings^1,2^. The balls of the ball bearing act as stress raisers which accelerate spall initiation process^3^ leading to costly consequences. Branch et al.^3^ observed that rolling elements in bearings can induce tensile stresses and localized damages and increase the probability for crack initiation and failure. The localized contact stresses arising from the ACBB have been found to be extremely large compared to those in rotating structural members such as shafts^4^. On the other hand, high contact stress can also result from the manufacturing error, unreasonable deformations and lubrication conditions^5,6^.

There are various research attempts in the area of contact stress analysis for bearings. This is because the contact stress phenomenon in rolling element bearings is critical and has been found to shorten the operative life of several mechanisms in engineering applications^7^. So far, there are four developments in the modeling and analysis of rolling bearings. These include static analysis, quasi-static analysis, quasi-dynamic analysis, and dynamic analysis. The current study considers the static analysis using Abaqus/standard (Dassault systems- version 2017).

The general limitations of theorical and experimental data has led to a number of numerical studies to understand the contact stress problem in bearings. By using FEA package various authors have developed models to predict the stress and displacement distributions in rolling element bearings^8–13^.

Since it is becoming more demanding for higher spindle speeds in engineering applications, one of the important issues among bearing manufacturers is to meet the requirement of high bearing performance. Studies have further indicated that operational temperatures will be expected to increase as a result of higher performance demanded of bearings. The high operational temperatures, as well as high-speed spindle rotations and other aggressive working conditions, to a great degree affect the bearing stiffness and stability.

The contact shape, size as well as stress magnitudes of ball bearings have been analyzed by using numerical tools based on ANSYS software^14–16^. The authors from these researches observed that use of simulation tools can provide useful data that can greatly improve bearing performance compared to theoretical techniques. Three- dimensional models of deep groove bearings were investigated by Zhaoping and Jianping^17^ to determine the contact stresses using ANSYS. It was shown that the accuracy of the results from the study was close to those derived from theoretical calculations. Some authors have analyzed bearing contact stresses using a combination of finite element (FE) and semi-analytical tools^18,19^. The studies showed that contact stresses as well as flexibility depended greatly on load magnitudes as well as distributions. Furthermore, it was shown that the experimental results were very consistent with those calculated from numerical results. The contact stress analysis of 3-D deep groove bearing models by Xin and Zhu^20^ using FE simulations has shown that FE can be beneficial in optimal design of bearings in addition to failure estimation making it a critical tool for use in real engineering applications.

From the various literature, it is noted that finite element analysis based on numerical simulation software such as ANSYS and ABAQUS have remarkable capabilities to predict the bearing design parameters including contact stresses and deformations. However, from the literature consulted, static FE modeling considering the effect of raceway thickness and housing stiffness on the stress behavior of the roller bearing has not yet been reported in previous works. This forms the novelty of this study.

Moreover, owing to the increasing demand for safer and more effective machine components, there is a requirement to design bearings with excellent functional features. In order to meet the challenging performance criteria imposed by the harsh operating conditions, new bearing designs are needed. Detailed investigations on the effect of contact stress are crucial towards realization of this goal.

In the present contribution, therefore, the stress behavior of the ACBB is investigated using FEA and also visualized using photoelasticity measurements. The understanding of the stress behavior of the ACBB will contribute to new designs as well as geometrical optimizations that will meet the industrial needs.

## Discretization of angular contact ball bearing

### Specification of angular contact ball bearing

**Figure 1:**
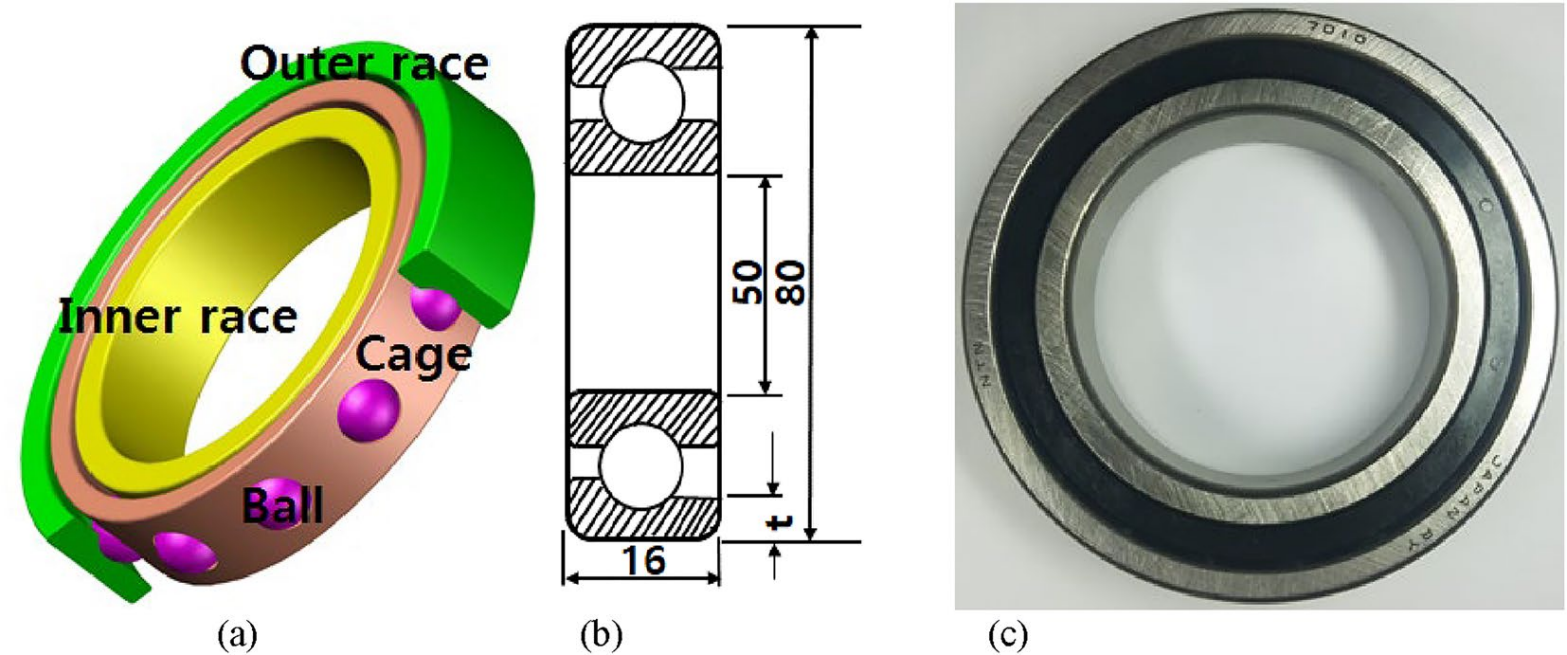
(**a**) Components of ACBB (**b**) cross-sectional view of ACBB (**c**) photo of actual bearing.

The angular contact ball bearing (ACBB) generally has two races with balls equally spaced in between the races. Balls are constrained by the cage so they can transfer load from inner race to outer race and vice versa. The basic components of the ACBB and **g**eneral description of the bearing including the size and photo, are shown in Fig. 1. The dimensions of the bearing in this study were; inner diameter 50 mm and outer race diameter 80 mm. The ball diameter was 8.7 mm while the bearing width was 16 mm. These dimensions are key during the 3D modeling of the ACBB. As shown in Fig. 1, the outer race thickness is indicated as t.

### Discretization of bearing parts

**Figure 2:**
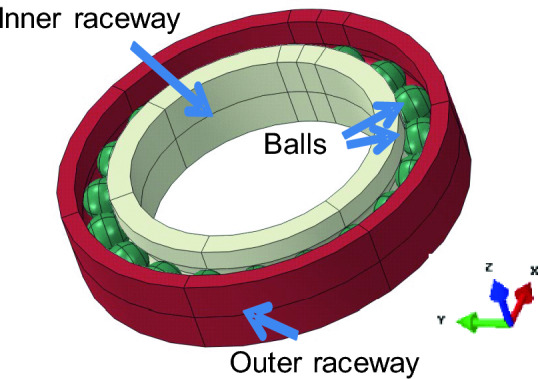
Solid model architecture of ACCB showing bearing balls, inner race, and outer race.

A three-dimensional solid model of the bearing body was created as shown in Fig. 2. For simplicity, the ball carrier/cage was not considered.

**Figure 3:**
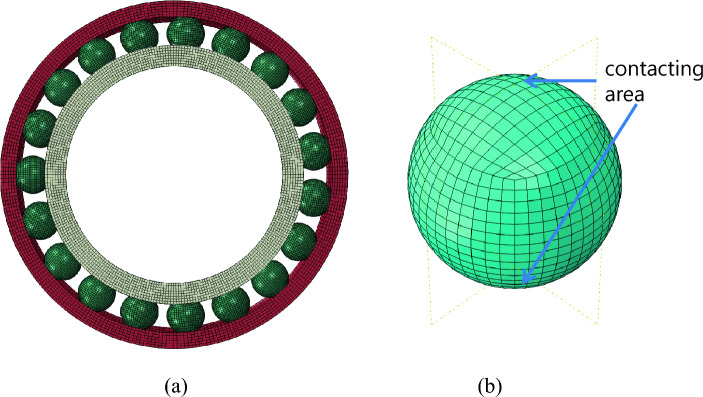
(**a**) Model of ACBB showing bearing balls, inner race and outer race (**b**) meshed ball showing contacting area.

A three-dimensional finite element analysis of the bearing was performed using Abaqus/standard software (Dassault systems- version 2017). Figure 3. shows the FEA model architecture. The bearing balls, inner race, and outer race were modeled as deformable bodies (three-dimensional linear hexagonal element, C3D8) as shown in Fig. 3a). Clearances between the ball and raceways are assumed to be zero. The spherical ball is meshed as shown in Fig. 3b), where the element shape of the contact surface is almost square to minimize the numerical errors. The number of meshes in the perimeter of the sphere was 48. The representative mesh size to model the contact phenomenon in the neighborhood of the contacting area was 0.54 mm. It is to be noted that although the mesh size of 0.54 may not be adequate, it is still within an acceptable range for qualitative analysis. The average total number of elements was 264,978.

## Effect of outer race thickness assembled into a rigid housing

### FEA model of ACCB with different outer race thickness

Stress fields in the raceway were evaluated numerically. The FEA model was established according to photoelastic experimental model^21^. The analysis using photoelasticity known as photoelastic experimental hybrid method (PEHM)^21^ considered the effect of outer raceway thickness on the stress behavior of the ACBB. By taking the smallest raceway thickness of 3.3 mm as the standard reference size, three conditions of outer raceway thickness were considered, namely; standard (T1, 3.3 mm), double (T2, 6.6 mm) and triple, (T3, 9.9 mm). The material for the shaft and housing in the experiment was aluminum, and during modeling they were assumed to be rigid bodies represented by analytic rigid surfaces. The material for the ball is steel with a Young’s modulus of E = 200 GPa. The inner raceway and outer raceway are made from epoxy whose value of Young’s modulus, E was taken as E = 15.6 MPa^22^

**Figure 4:**
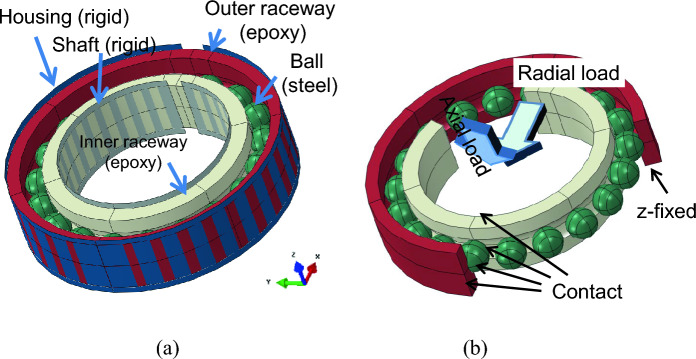
(**a**) Three-dimensional solid model of bearing with global coordinate system and (**b**) the contact, load, and boundary conditions.

Figure 4 shows the full model of 3D image of the bearing with the various components (balls, housing, inner and outer raceways and shaft). The outer race thickness was T1 and both axial load and radial load were applied. For the numerical convergence, the axial load was applied first, and then radial load was applied subsequently. A radial force of 30 N was applied to the ‘reference point’ of the shaft (analytic surface) while an axial force was applied to another ‘reference point’ kinematically coupled to the upper side of the inner race where the axial load was applied. Small sliding contact conditions were applied to all contacting surfaces as indicated in Fig. 4b; between the shaft and inner race, between the housing and outer race, between the ball and inner race, and between the ball and outer race. The friction coefficient was assumed to be μ = 0.02. To eliminate the rigid body motion of balls, gravitational force was applied in the z-coordinate. As a result, corresponding frictional forces are engaged, and the spherical ball can remain stable between the inner and outer race without the ball carrier which exists in the actual bearing.

### Stresses and contact pressures

**Figure 5:**
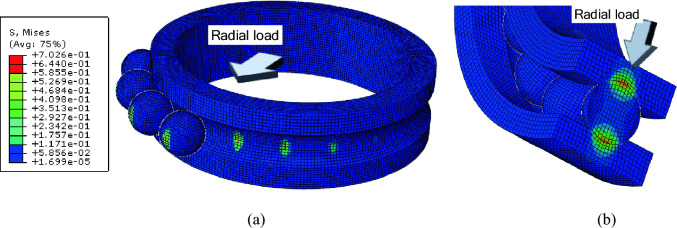
Contour plot of von Mises stress at (**a**) inner raceway and (**b**) cross-sectional area.

In this case, a radial displacement of 0.1 mm was applied and in Fig. 5, contour plots of the von Mises stress distribution on the inner and outer raceways of ACBB are displayed. Von Mises stress is serve as a measure to predict yielding of materials under the loading conditions. In the context of ball bearings in this study, von Mises stress and distribution was measured to determine the stress behavior of the ACBB when loaded. Of all the balls, only the 9 balls placed in the direction of the force are observed to contribute to the load support. The stress is highest at the centrally located ball and decreases as one moves away from the central ball (Fig. 5a). It is observed that the von Mises stress on the inner raceway is higher (0.7 MPa) than that on the outer raceway (0.64 MPa) (Fig. 5b). This corroborates well with the experimental results which showed that inner raceway stresses were higher than outer raceway stresses^21^.

**Figure 6:**
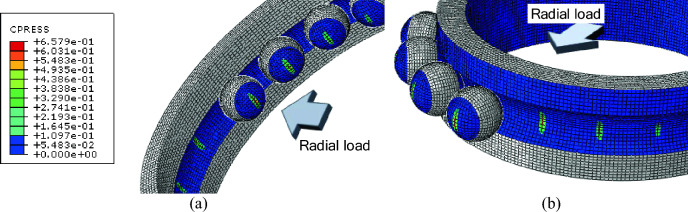
Contour plot of contact pressures at (**a**) outer raceway and (**b**) inner raceway.

Figure 6 which shows the contact pressures within the angular contact ball bearing (ACBB) indicates that the highest pressure is experienced by the ball located at the center. Interestingly, like the von Mises stresses, contact pressure gradually decreases as you move away from the center towards the outer balls.

### Stresses with respect to the thickness of outer raceway

**Figure 7:**
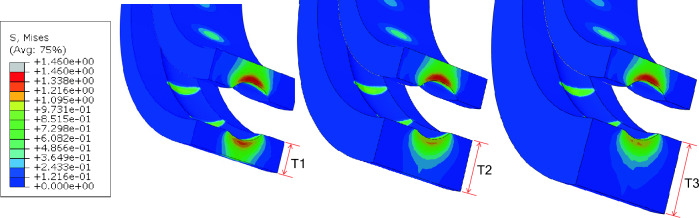
von Mises stress contour plot in raceways (**a**) standard T1, (**b**) double T2, and (**c**) triple T3.

The variation of stresses with respect to the thickness of outer raceway was observed. The thickness was changed by extending the radius. In this analysis, radial load of 30 N was applied. Contour plots of von Mises stress fields for the three thickness levels are shown in Fig. 7. For clarity, steel balls are hidden, and only inner raceway and outer raceway are shown. The stresses on the outer race are observed to decrease with increasing thickness and was minimum when t = T3. Deformations on outer race with thickness t = T1 were found to be large and only small deformations were observed on outer race with thickness of t = T3. Therefore, from these results it is shown that a larger outer race thickness can decrease the stresses as well as deformations and hence provides an important guideline for bearing designs.

**Figure 8:**
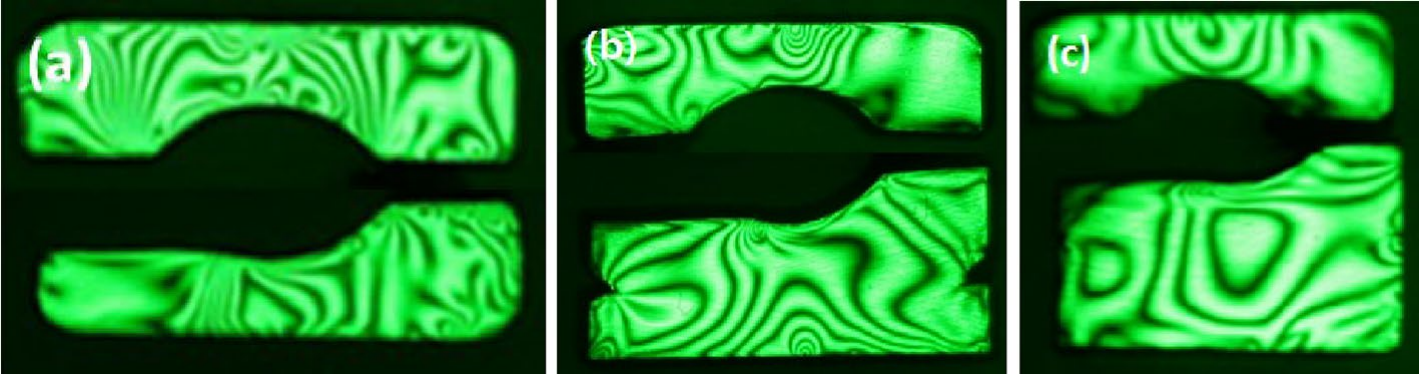
Isochromatic stress fringes in raceways from photoelasticity (**a**) standard T1 (**b**) double T2, and (**c**) triple T3.

**Figure 9:**
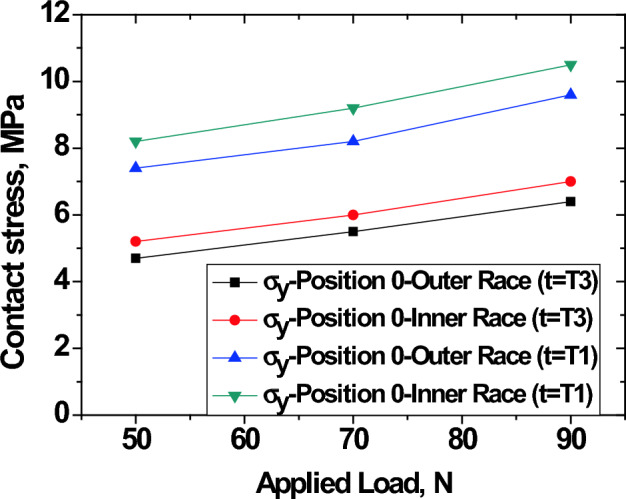
Variation of contact stress with applied load and race thickness ^21^.

Experimental results showing isochromatic stress fringes in raceways from photoelasticity are shown in Fig. 8. Also, Fig. 9 has been extracted from experimental data to show the changes in contact stress, σ_y_, on the inner and outer races at position 0 based on thickness, namely; smallest (t = T1) and largest (t = T3)^21^. It is confirmed that the experimental results are in agreement with FEA results in Fig. 7. Both experimental and simulation results indicate that the stress on the outer race was decreased with increasing thickness. This is because the thicker outer race has enough volume to absorb external load transferred from balls. Therefore, outer raceway thickness has remarkable influence on stress concentrations and distributions of the bearing under consideration. This will serve as good guidance for bearing design and geometrical optimization.

## Effect of the race thickness assembled into a pillow block housing

### FEA model of bearing in housing

**Figure 10:**
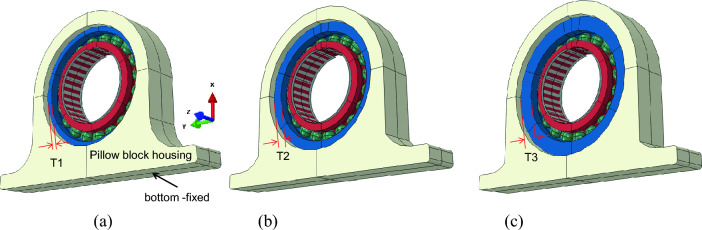
Three-dimensional model of ACBB mounted in typical pillow block housing. (**a**) t = T1 (3.3 mm), (**b**) t = T2 (6.6 mm) and (**c**) t = T3 (9.9 mm).

The effect of race thickness was studied when it was applied to deformable pillow block housing. To achieve this, the ACBB was assembled into an actual housing and evaluated by FEA. The bearing material was assumed to be a steel with an elastic modulus of 200 GPa and a Poisson’s ratio of 0.33. The housing is a typical commercial pillow block housing. Figure 10 shows a 3D image of assembled bearing set for t = T1, T2 and T3. Mesh size the bearing was the same as the model in section “Discretization of bearing parts”, and a housing part was added with a small sliding contact condition, resulting in a total number of elements of 283,612 in the T1 model. In this model, the effects of thickness of outer race, loading direction, and stiffness of housing were studied. A radial force of 400 N and axial force of 20 N were applied. The thicknesses of outer race were the same as those previously described, namely; t = T1 (3.3 mm), T2 (6.6 mm) and T3 (9.9 mm). Housing was considered deformable with the bottom fixed.

### Stress distribution in a bearing with respect to loading direction and housing stiffness

**Figure 11:**
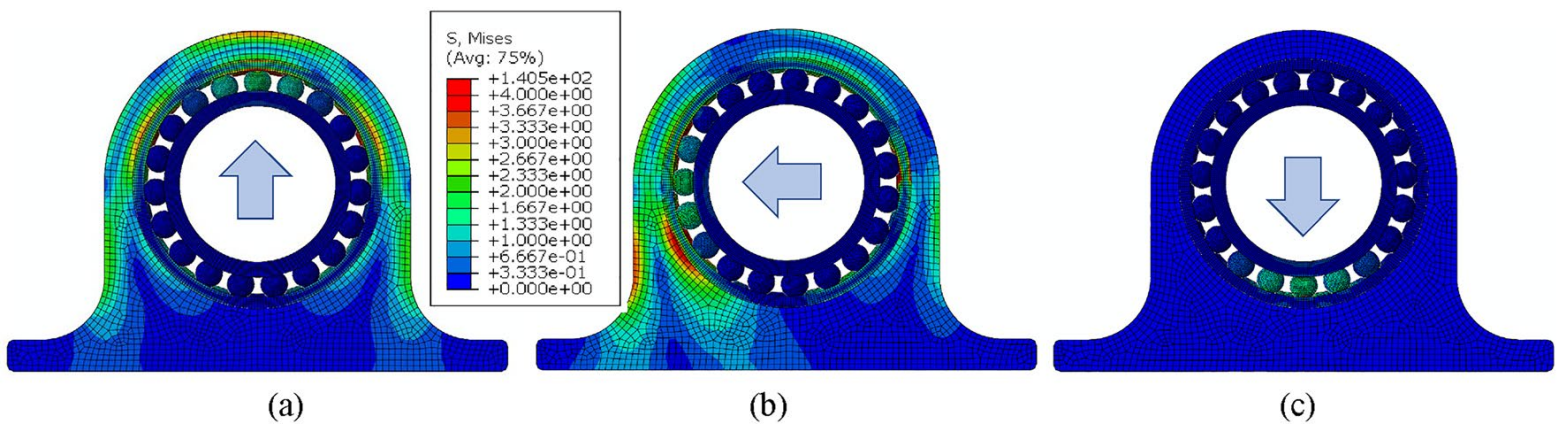
Contour plot of von Mises stress. (**a**) UPWARD, (**b**) LEFTWARD, and (**c**) DOWNWARD.

The housing has representative variation of shape; with a thin upper side, thick lower side, and transient left and right side. This variation may show different responses to radial load during operation. Therefore, three directions of radial loading were considered in the analysis of stress distributions, namely; UPWARD, LEFTWARD and DOWNWARD. Figure 11 shows von Mises stress distributions in ACBB with respect to the loading directions when assembled in the bearing housing. As indicated in the stress legend, the maximum stress was found to be 140 MPa. In addition to varying the loading direction, the housing stiffness was also varied and the stress magnitudes were measured.

The effect of housing stiffness was indirectly verified by changing the elastic modulus of housing part. Three different values of modulus of elasticity were considered; namely; SOFT (100 GPa), NORMAL (200 GPa), HARD (300 GPa). It is to be noted that the elastic modulus of bearing components was 200 GPa.

**Figure 12:**
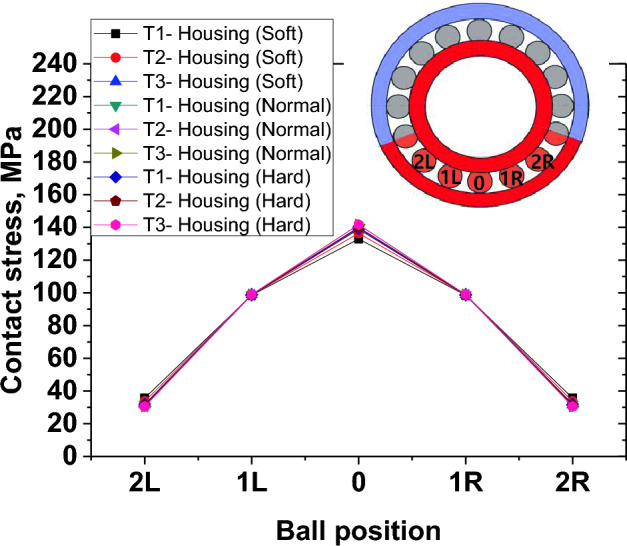
Variation of contact stress distributions among balls along circumference according to housing stiffness and UPWARD loading direction.

Stress magnitudes were extracted from the balls at various ball positions along the circumference as shown in Fig. 12. The results from Fig. 12 show the stress magnitudes and distributions when the loading direction was in UPWARD and housing stiffness was SOFT, NORMAL and HARD. Ball positions are inset as 0,1R, 2R, 1L and 2L, etc. Position 0 ball is lowest position while R indicates balls located to the right of zero position and L denotes balls located on the left side of the zero position. The highly loaded balls were; 0,1R, 2R, 1L and 2L. The stress magnitude is highest at location 0 and decreases in the subsequent balls on either sides. It was found that when the loading direction was DOWNWARDS, the stiffness does not have a pronounced effect on stress values (Fig. 11c).

When the load was applied in the UPWARD direction of the soft housing, highest contact stress was 132.93 MPa while the maximum contact stress was 146.5 MPa when load was applied in DOWNWARD direction. Thickness of outer race was t = T1. For normal housing, stress values of 138.98 MPa and 139.83 MPa were obtained from UPWARD direction when race thickness was t = T1 and t = T3 respectively.

**Figure 13:**
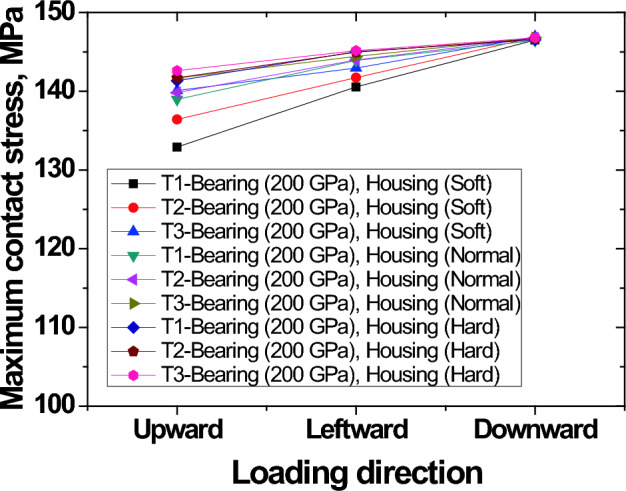
Variation of maximum contact stress according to loading direction, race thickness and housing stiffness.

A summary of how contact stress varies with direction of load application, race thickness and housing stiffness is shown in Fig. 13. Both direction of loading and race thickness were found to have some effect on contact stress. UPWARD direction of loading gives stress values lower than stress values in DOWNWARD direction.

From the results, race thickness and direction of load application seem to influence stress concentrations. Ratio of raceway thickness of 1:1 has lowest stress values. Ratio of 2:1 has a higher stress while ratio of 3:1 has the highest stress. The observation of increase in stress due to increase in thickness ratio is confirmed by direction of load application. When load direction is applied in the direction where thickness of outer race is thickest, stress values are highest.

Low contact stress values are recorded for UPWARD loading direction and increase almost linearly. It is interesting to note that for all cases of race thickness and housing stiffness, the value of stress at DOWNWARD direction is constant.

### Deformation of outer race with respect to loading direction and housing stiffness

**Figure 14:**
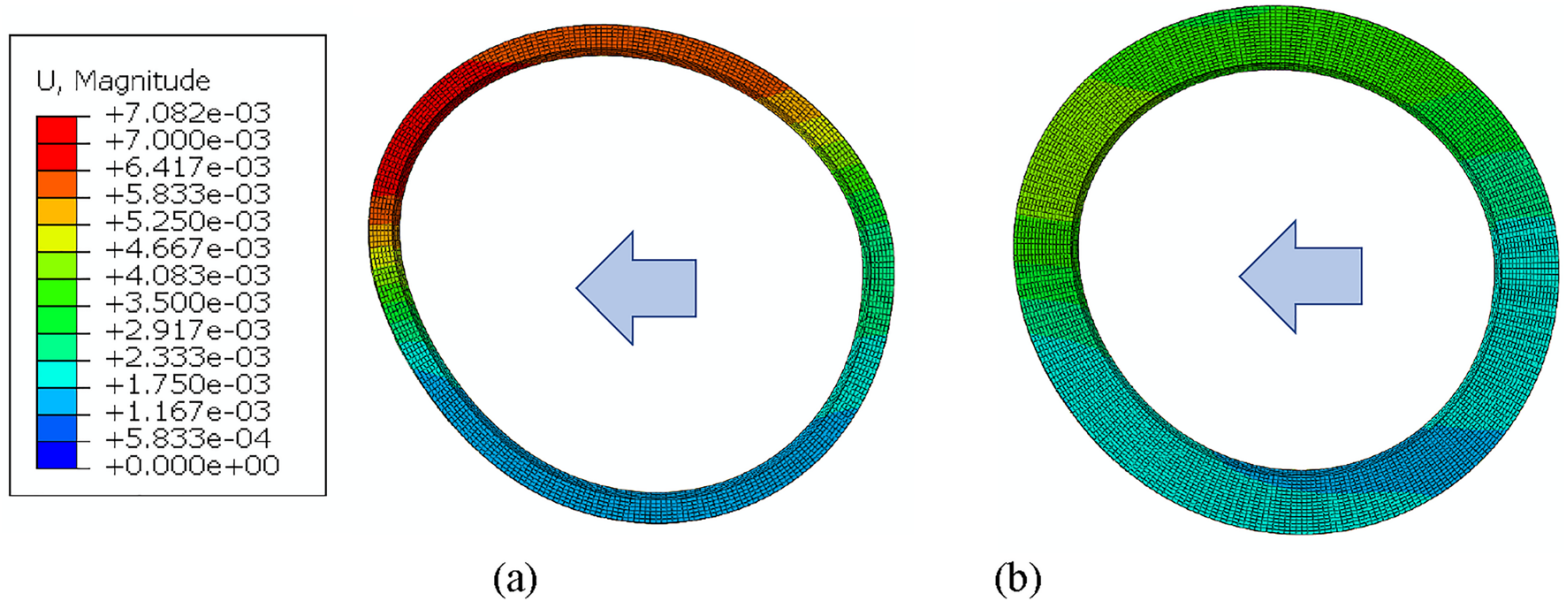
Deformation of outer race (**a**) t = T1 (**b**) t = T3 for soft housing (loading direction LEFTWARD, magnification factor 1,000).

**Figure 15:**
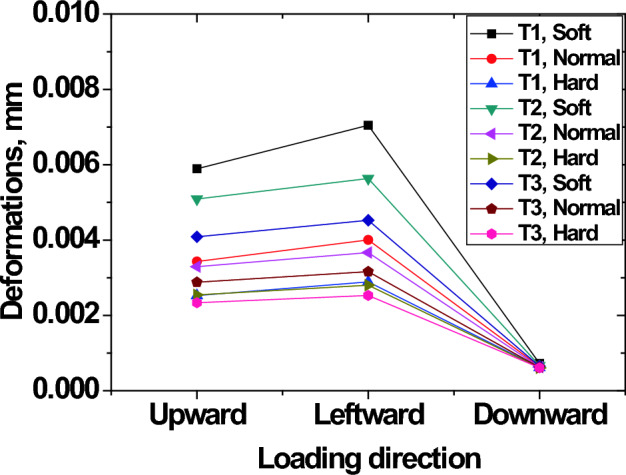
Outer race deformations according to loading direction, race thickness and housing stiffness.

The deformations occurring on the outer race are shown in Fig. 14 when the bearing housing stiffness was SOFT for two thickness levels of t = T1 and t = T3. Results summarizing the variation of deformations on outer race according to loading direction, race thickness and housing stiffness are given in Fig. 15. It is generally shown that deformations on outer race with t = T1 thickness were large ($7.08\times {10}^{-3}$ mm) compared to those on race with t = T3 ($2.33\times {10}^{-3}$ mm). Large deformations induce higher shear stresses on thin races than on thick races. Many failure mechanisms agree that high shear stresses originating from the subsurface are extremely harmful^23^. These stresses can cause spalling in bearings and propagates into a network of cracks^23^.

### Correlating race thickness to bearing life

Contact stresses play an important role in determining the operational life of machine parts such as gears and bearings. The bearing performance can be estimated by bearing life Eq. (1)^4^. In bearings technology, bearing life is the number of revolutions a bearing undergoes until flaking (peeling off of the surface) occurs. Guillermo et al.^24^ noted that, the performance of bearings is based on shear stress originating from the contact surfaces. Since large deformations occur in outer race with smallest thickness, it is expected that the shear stresses will be highest in thin races. Recently, Mose et al.^22^ noted that shear stress is considered in engineering stress models as the most damaging as it controls crack initiation. As a result of the observation of large deformation on thin race of $7.08\times {10}^{-3}$ mm compared to that on thick race of $2.33\times {10}^{-3}$ mm, it can be deduced that thin bearing race is more prone to failure compared with thick bearing races.

1 $${L}_{10}={\left(\frac{C}{P}\right)}^{p},$$

where C = basic dynamic load rating (N), P = equivalent dynamic load (N) and p = exponent defined by the bearing type.

## Conclusions

A finite element analysis was done using Abaqus/standard (Dassault systemes). The following conclusions have been derived from this study.

1. Inner race has high stress magnitudes compared to outer race. This agrees well with experimental data
2. Through FEA, balls with high stress concentrations have been identified and contact stress magnitudes extracted.
3. Contact stress extracted from FEA results demonstrated that the outer race with t=T1 does not necessarily lead to much higher stress compared to race with t=T3. Instead, race with t=T3 led to slightly higher stresses.
4. FEM results showed that direction of load application has an influence in the contact stresses.
5. Location of the ball at any instant determines stress magnitude. Regardless of the loading direction, race thickness and housing stiffness, the heavily stressed balls were located at positions 0, followed by 1R, 1L, 2R, 2L.

## Data Availability

The datasets used and/or analyzed during the current study are available from the corresponding author on request.
